# Combination of serum matrix metalloproteinase-3 activity and EBV antibodies improves the diagnostic performance of nasopharyngeal carcinoma

**DOI:** 10.7150/jca.46977

**Published:** 2020-08-18

**Authors:** Yiqiu Li, Zhibo Feng, Shan Xing, Wanli Liu, Ge Zhang

**Affiliations:** 1 Department of Microbial and Biochemical Pharmacy, School of Pharmaceutical Sciences, Sun Yat-sen University, No.132 Waihuandong Road, University Town, Guangzhou 510006, China.; 2 Department of anatomy, Xinxiang Medical University, Xinxiang, Henan 453700, China.; 3 State Key Laboratory of Oncology in South China, Collaborative Innovation Center for Cancer Medicine, Guangdong Key Laboratory of Nasopharyngeal Carcinoma Diagnosis and Therapy, Sun Yat-sen University Cancer Center, Guangzhou 510060, China.

**Keywords:** matrix metalloproteinase-3 (MMP3), cancer metastasis, enzymatic activity, nasopharyngeal carcinoma diagnosis, tumor serum marker

## Abstract

**Objective:** Nasopharyngeal carcinoma (NPC) is a malignant head and neck tumor that is highly prevalent in Southeast Asia. The two traditional NPC markers VCA-IgA (EBV viral capsid antigen) and EA-IgA (EBV early antigen) are limited in the screening and diagnosis of NPC. The purpose of present study is to evaluate the diagnostic value of matrix metalloproteinase-3 (MMP3) in NPC.

**Methods:** The levels of 23 secretory MMPs in serum samples from 15 healthy controls and 26 NPC patients were detected by Cytokine Antibody Array 2000. Immunohistochemistry, Real-time PCR and western bolt were used to detect MMP3 mRNA and protein levels in NPC tissues and cell lines. The serum protein levels of MMP3 were further measured by ELISA in healthy control individuals (n = 200) and NPC patients (n = 206).

**Results:** MMP3 can be expressed and secreted by both NPC and fibroblast cell lines, suggesting that the higher expression of MMP3 protein in both tumor nests and stromal of NPC tissues may be the source of circulating MMP3 in NPC patients. Furthermore, we found out both MMP3 concentration and enzymatic activity were significantly increased in the NPC group (n = 206) than the healthy control group (n = 200) (*P* < 0.001). However, serum MMP3 enzymatic activity, but not MMP3 concentration, was significantly associated with the progression of NPC. In addition, serum MMP3 activity was more valuable in diagnosis of NPC than its concentration (0.86 *vs.* 0.78, AUC), and MMP3 activity can improve the diagnosis of NPC by combining with EBV-infection biomarkers VCA-IgA and EA-IgA with a sensitivity of 91.5% and a specificity of 92.3%.

**Conclusions:** This study suggested the combination of MMP3 activity and EBV antibodies may be a useful biomarker for screening and diagnosis of NPC.

## Introduction

Nasopharyngeal carcinoma (NPC) is a malignant head and neck tumor with a distinct racial and geographical distribution that is highly prevalent in Southeast Asia 1. Due to the anatomical location and mild early symptoms, the diagnosis of NPC in the early stage is difficult. Although VCA-IgA (EBV viral capsid antigen) and EA-IgA (EBV early antigen) were used as common screening and diagnosis markers for NPC, the lack of specificity of the VCA-IgA assay and sensitivity of the EA-IgA assay limits their effectiveness in NPC diagnosis 2,3. Therefore, there is an urgent need to explore valuable screening biomarkers for NPC patients.

Matrix metalloproteinases (MMPs) belong to a zinc-dependent family of endopeptidases, which displays a broad ability to degrade extracellular matrix 4. MMPs family plays a role in a wide range of tumorigenesis, including early carcinogenesis events, tumor growth, tumor invasion and metastasis 5. Among the family of MMPs, MMP3 is classified as a stromelysin and is thought to involve the process of metastasis for its ability to cleave a variety of matrix protein substrates such as collagen types II, IV, IX, X, and XI, fibronectin, gelatins, proteoglycanase and E-cadherin 6. *In vivo*, MMP3 is secreted as inactive zymogens which would be activated when cleaved by proteolytic enzymes like trypsin-2 7. Interestingly, MMP3 have been demonstrated to over-express in various human tumor tissues. Recently, MMP3 have been considered as potential diagnostic or prognostic biomarkers in some types of cancer, especially in head and neck squamous cell carcinoma 8-11.

In the present study, elevated protein levels of MMP3 were observed in serum samples from 26 NPC patients compared with 15 healthy controls based on 23 secretory MMPs Array. Furthermore, we measured MMP3 protein level and enzymatic activity in serum from NPC patients and investigated the associations of MMP3 concentration and its activity with patients' clinical outcomes to assess whether serum MMP3 is a valuable diagnostic biomarker for NPC.

## Materials and Methods

### Patients, blood and tissue samples

Serum from 26 NPC patients and 15 healthy volunteers were obtained in October 2015 and used for Cytokine Array. Serum samples were stored at 80°C and were measured within 3 months.

Sera used for MMP3 arrays were obtained from 206 patients with NPC between March 2016 and August 2016. The cohort consisted of 153 male patients and 53 female patients. Patients ranged in age from 16 to 75 years (mean: 44.7 years). All sera were collected from NPC patients at the time of diagnosis. The characteristics of the 206 patients are described in Table 1.

Sera from 200 healthy volunteers (149 males, 51 females) with ages ranging from 19 to 77 years (mean: 45.1 years) were collected and used as controls. Healthy controls were selected from an archive of blood samples, and the control samples were matched as closely as possible to the NPC group with respect to previous handling and the time period of sample collection.

A 5 mL blood sample from each participant was allowed to clot for 30 to 60 min at room temperature. Each clotted sample was centrifuged at 1,500 g for 10 min. All sera were then aliquoted and frozen at -80°C until use. Enzymatic activity test was conducted within 24 h after sample collecting.

We obtained paraffin-embedded tumor tissue samples of NPC from 15 patients who were diagnosed clinically before chemoradiation between May and December of 2013. None of the patients had received anticancer treatment prior to surgery, and all of the patients had histologically confirmed primary NPC in this retrospective study.

All of the blood and tissues samples were collected at the Cancer Center of Sun Yat-Sen University. The study was approved by the Ethics Committee of Sun Yat-Sen University Cancer Center and informed consent was obtained from each patient.

### Cytokine Array

A Human Cytokine Antibody Array (Series 2000, RayBiotech, Norcross, Georgia, USA) was used to screen MMPs in NPC, which analyzed 23 MMPs related to angiogenesis, immunity and tumor proliferation pathways. Samples were analyzed according to the manufacturer's instructions. Two replicates per antibody were used to increase the accuracy, and the coefficient of variation (CV) was less than 10%.

### Cell culture

NPC cell lines CNE1 (EBV-negative), C666-1 (EBV-positive), and human pulmonary fibroblast cell line MRC5 were cultured in RPMI 1640 medium (Gibco, CA, USA). All of the cell lines were maintained in our laboratory, and all media were supplemented with 10% fetal bovine serum (Gibco, Brazil).

### RNA preparation and quantitative real-time PCR

Total RNA was extracted from NPC and fibroblast cell lines using RNAiso Plus (TaKaRa, Dalian, China) according to the manufacturer's instruction. Reverse transcription of total RNA (2 μg) was performed using SuperScript II reverse transcriptase (GIBCO BRL, Grand Island, NY, USA). The primers used for real-time RT-PCR were as follows: MMP3: forward 5′- AGTCTTCCAATCCTACTGTTGCT -3′ and reverse 5′- TCCCCGTCACCTCCAATCC -3′; GAPDH: forward 5′-GCACCGTCAAGGCTGAGAAC-3′ and reverse 5′-TGGTGAAGACGCCAGTGGA-3′.

### Western blot

Total protein was extracted using a lysis buffer and protease inhibitor (Beyotime Biotechnology, Shanghai, China). Equivalent protein amounts were denatured in an SDS sample buffer and then were separated by SDS-PAGE and transferred onto polyvinylidene difluoride membrane. After being blocked with 5% non-fat dry milk in PBS containing 0.05% Tween-20, the blotted membranes were incubated with anti-MMP3 antibodies, (1:1000, bioworld, MN, USA) and then incubated with a secondary antibody (1:5000, Boster, Wuhan, China). GAPDH protein levels were also determined by using the specific antibody (1:1000, Boster, Wuhan, China) as a loading control.

### Immunohistochemistry

Formalin-fixed, paraffin-embedded NPC sections were incubated with anti-MMP3 antibody (1:100, bioworld, MN, USA) overnight at 4°C. After washing in PBST, the tissue sections were treated with a horseradish peroxidase-conjugated anti-goat secondary antibody (1:1000, Zymed, CA, USA). The tissue sections were then developed with 3-diaminobenzidine tetrahydrochloride for 10 seconds, followed by counterstaining with 10% Mayer's haematoxylin. The degree of staining was reviewed by two independent observers.

### ELISA

Serum MMP3 protein concentrations were determined by double-antibody sandwich ELISA according to the manufacturer's instructions (SMP300, R&D systems, MN, USA). Briefly, 100 µl/well of test serum or standard was added in 96-well plates and incubated them for 2.5 h. Subsequently, 100 µl/well of the biotinylated antibody solution was added and incubated for 1 h. Next, 100 µl/well of Streptavidin solution was added and incubated for 45 min. Finally, 100 µl/well of TMB One-Step Substrate Reagent was added, and the reaction was stopped by addition of stop solution; the absorbance was measured at 450 nm. Each test included a standard control (CV < 10%).

### MMP3 specific activity Array

Serum MMP3 specific activity was determined by MMP3 fluorescence-activated substrates 12 (DNP-PYAYWMR, Chinese peptide company, Hangzhou, China). The internally quenched peptide substrate (Dnp-PYAYWMR) that, upon cleavage by MMP-3, produces the products, Dnp-PYA (quiet) and YWMR (a fluorophore at 360 nm). Briefly, test serum was diluted 5-fold in PBS (pH = 7.4), and 100 μL of the diluted serum and 50 μL substrates (50 μM, dissolved in PBS/DMSO = 7/3, v/v) (9) was added and further incubated for 2 h at 37 °C avoiding light. After incubation, MMP3 fluorescence intensity was measured by Multifunctional enzyme mark instrument (Flex Station 3, Molecular devices, USA). Read Ex/Em = 328/350 nm. The RFU of fluorescence generated by hydrolysis of the substrate is ∆RFU = R_350_ - R_328_. There are three repeats for each test.

### Statistical analysis

Statistical analyses were performed with the SPSS 20.0 (SPSS Inc.) The relationships between serum MMP3 protein levels or activity and the clinicopathologic features were analyzed by the Mann-Whitney U test. The comparisons of MMP3 concentration or activity among different groups were assessed using the Kruskal-Wallis test. The efficacy of MMP3 protein levels or activity was evaluated by the area under receiver operating characteristic (ROC) curve (AUC). The cut-off value for MMP3 concentration or activity was defined as the value with the maximization of the Youden index. Furthermore, sensitivity and specificity were used to compare the efficiency of diagnosis among MMP3 protein levels, MMP3 enzymatic activity, EA-IgA and VCA-IgA. All statistical tests were two-sided, and *P* < 0.05 was considered statistically significant.

## Results

### Identification of the potential serum MMPs markers in NPC

**Figure 1:**
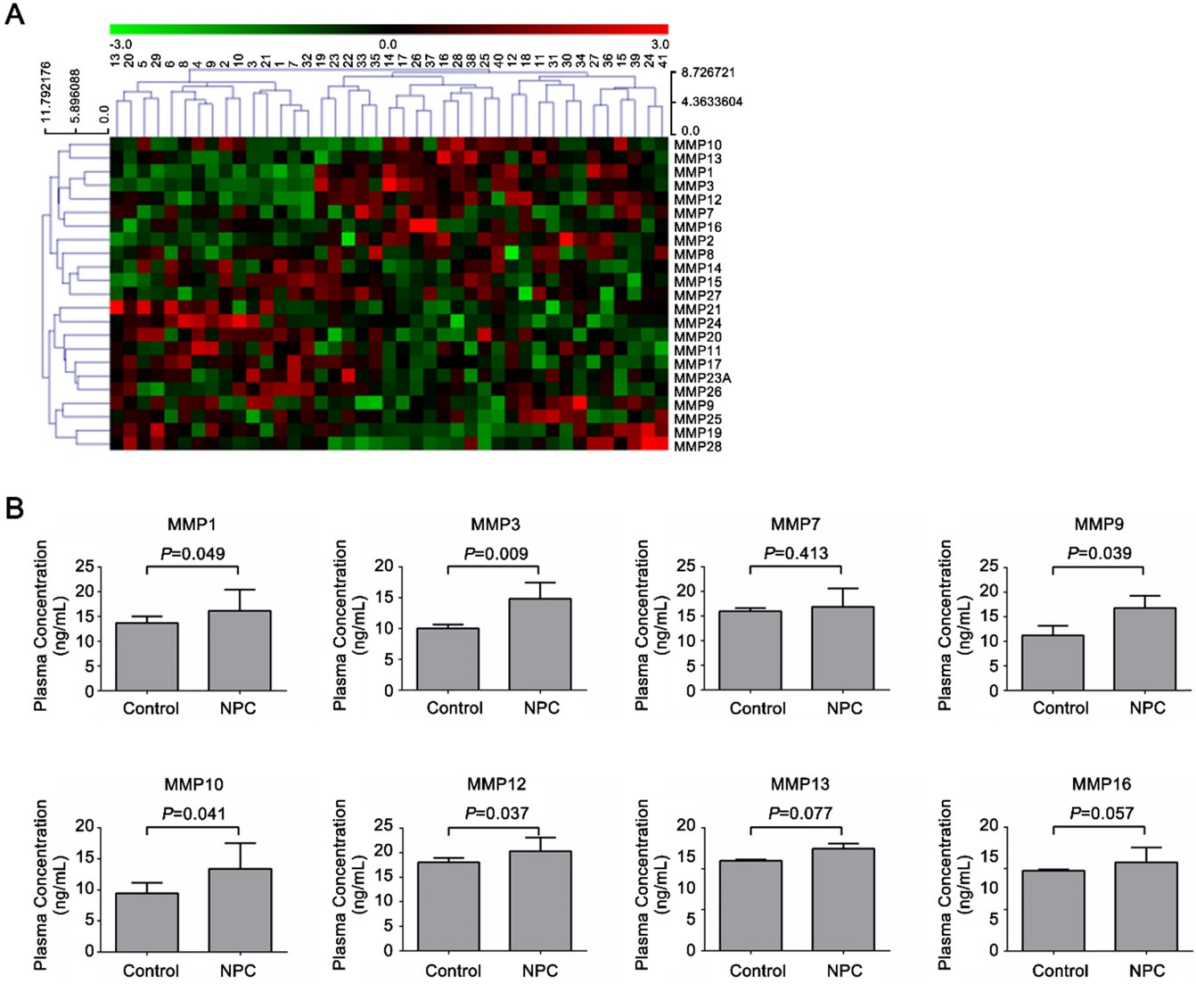
** Characterization of MMPs levels in NPC patients and healthy controls.** Serum levels of MMPs were analyzed by antibody-based cytokine microarray. (**A**) Cluster analysis of 23 MMPs proteins (MMP1, 2, 3, 7, 8, 9, 10, 11, 12, 13, 14, 15, 16, 17, 19, 20, 21, 23A, 24, 25, 26, 27, 28) and (**B**) Elevated protein level of 8 MMPs (MMP1, 3, 7, 9, 10, 12, 13, 16) in 15 healthy controls and 26 NPC patients.

To screen the potential diagnostic markers in NPC, the levels of 23 secretory MMPs (MMP1, 2, 3, 7, 8, 9, 10, 11, 12, 13, 14, 15, 16, 17, 19, 20, 21, 23A, 24, 25, 26, 27 and 28) in serum samples from 15 healthy controls and 26 NPC patients were detected by Human Cytokine Antibody Array 2000. Cluster analyses of 23 MMPs proteins were shown in Figure 1A, and elevated protein levels of 8 MMPs were shown in Figure 1B. Among the all secretory MMPs, the serum levels of MMP1, 9, 10 and 12 showed a significant difference between the two groups at *P* < 0.05, and MMP3 levels exhibited significant difference at *P* < 0.01.

### Characterization of MMP3 expression in NPC tissues and NPC cell lines

**Figure 2:**
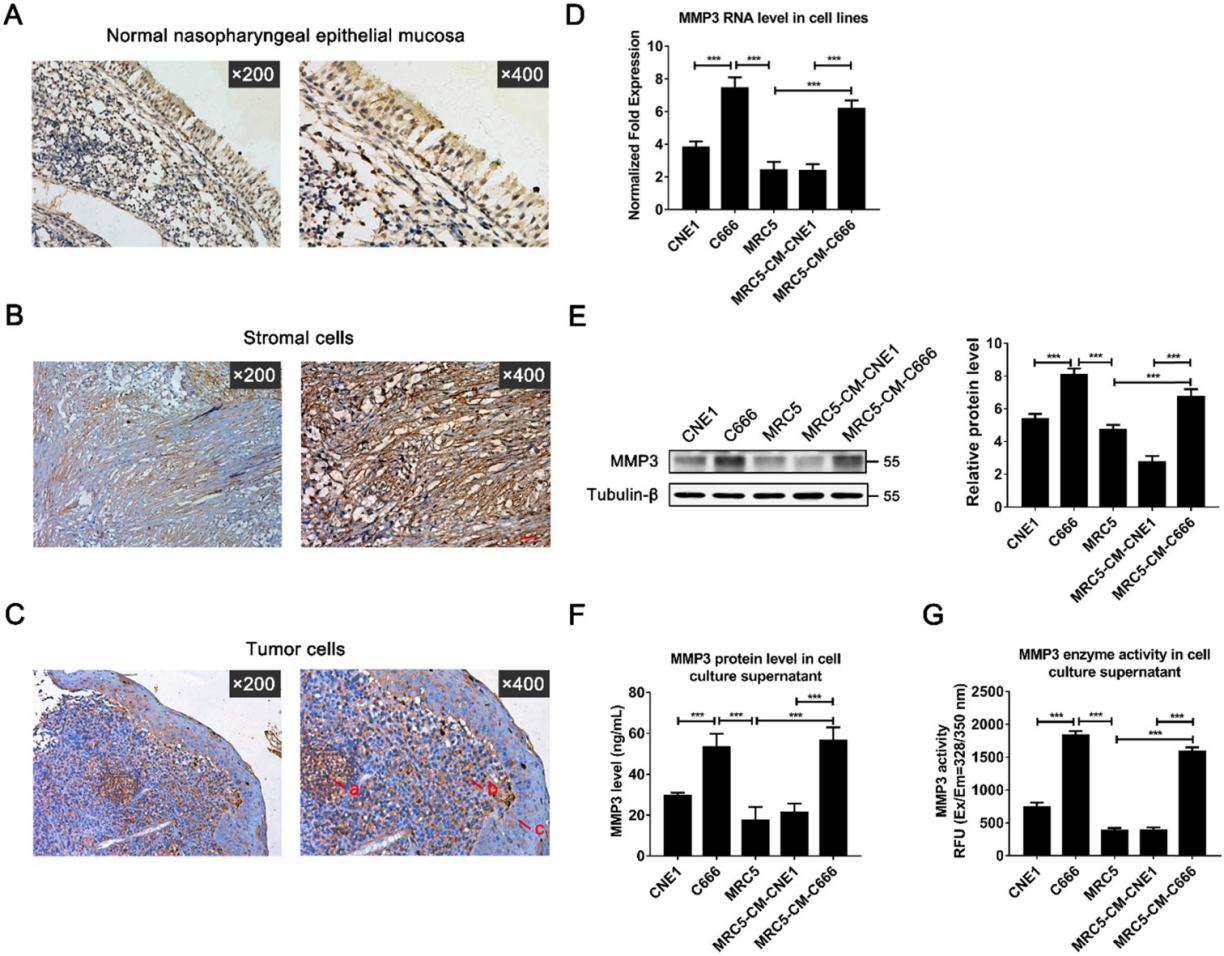
** Expression of MMP3 in NPC tissues and cell lines.** (**A**) The normal nasopharyngeal epithelial mucosa (×200; ×400). (**B, C**) Expression of MMP3 in stromal cells (×200; ×400) and tumor cells (×200; ×400) was detected by immunohistochemical staining in tumor tissues from patients with NPC. (Arrows in C mean a) lymphocytes; b) tumor cell; c) metaplastic cells). (**D**) MMP3 mRNA expression of CNE1 cell, C666 cell, MRC5 cell, MRC5 cultured with CNE1-conditioned culture (CM) and MRC5 cultured with C666-CM were detected by qRT-PCR; (**E**) MMP3 protein expression was detected by western blotting. (**F**) Secretory MMP3 concentration was detected by ELISA and (**G**) enzymatic activity was detected by fluorescence-activated substrate analysis. Data are shown as means ± SD. *** *P* < 0.001, n=3.

To further investigate the exact expression state and location of MMP3 *in vivo*, the protein levels of MMP3 were determined by immunohistochemistry in 15 paraffin-embedded archived NPC tissues (Figure 2A-C). MMP3 protein was detected in all of the 15 NPC tissues (100%). Strong MMP3 staining was mainly observed in both of tumor stroma (Figure 2B) and cancer cell nests, and the weak staining was observed in some areas of metaplasia with atypical hyperplasia (Figure 2C). Weak MMP3 staining was observed in normal nasopharyngeal epithelial cells (Figure 2A). Moreover, MMP3 straining was presented in carcinoma cells, NPC tissue fibroblasts-like cells and a few lymphocytes. The major localization of MMP3 was observed in the cell membrane or distributed between the cytoplasm (Figure 2B, C).

Furthermore, Real-time PCR and western blot analysis showed that both MMP3 mRNA and protein were expressed differently in two NPC cell lines: moderately in CNE1 cell line which is EBV negative, strongly in C666 cell line which is EBV positive (Figure 2D, E). Besides, MMP3 also presented weak expression and activity in fibroblasts cell line MRC5. Moreover, MRC5 cell line presented high MMP3 levels after co-culturing for 72 h with the conditioned medium (CM) of C666 cell line, but not with the CM of CNE-1 (Figure 2D, E). ELISA and fluorescence-activated substrate analysis showed that both MMP3 protein level and specific activity were higher in the culture supernatant of C666 and MRC5-CM-C666 cell (Figure 2F, G). These results indicated that MMP3 is widely expressed in the tumor and stoma of NPC tissues and the expression of MMP3 in NPC patients may be closely related to EBV.

### Diagnostic value of serum MMP3 concentration in NPC

**Figure 3:**
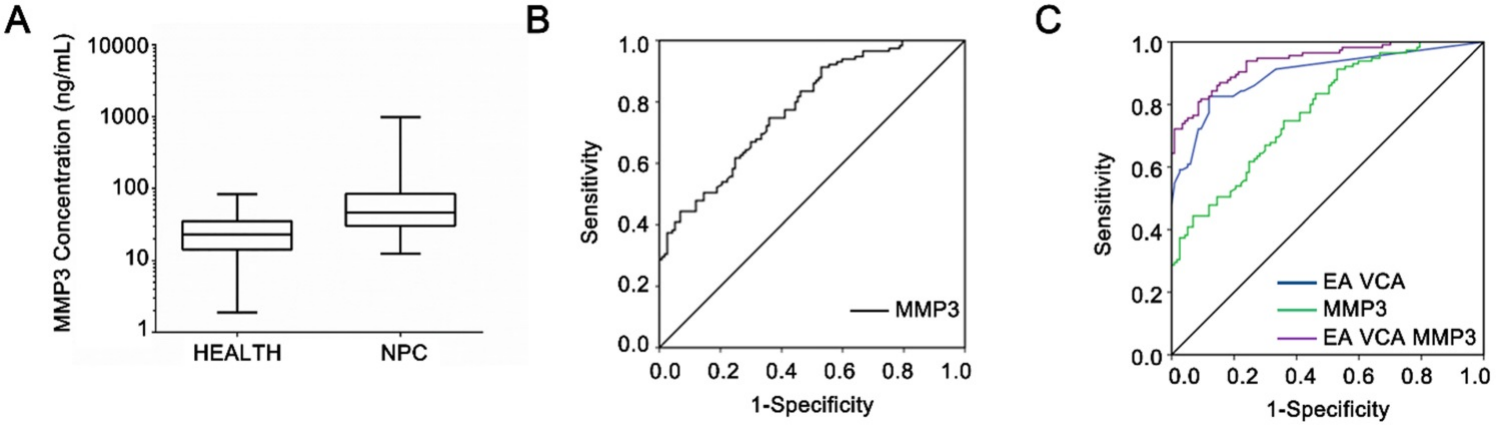
** Diagnosis value of serum MMP3 concentration.** (**A**) Serum MMP3 concentrations in NPC and control groups are plotted as a distribution. (**B**) ROC curves of the serum MMP3 protein levels. The estimated area under the ROC curve was observed as AUC = 0.78. (**C**) ROC curves for the diagnostic strength to identify NPC using MMP3 concentration, EA-IgA and VCA-IgA. (EA-IgA+VCA-IgA: AUC = 0.89; EA-IgA +VCA-IgA +MMP3: AUC = 0.93).

The serum protein levels of MMP3 were measured by ELISA in healthy control individuals (n = 200) and NPC patients (n = 206), respectively. As shown in Figure 3A, serum MMP3 concentrations of patients with NPC (185.97 ± 432.21 µg/L) were significantly higher than those of the healthy controls (33.95 ± 30.58 µg/L, *P* < 0.001).

Next, the ROC curve was plotted to identify the optimum diagnostic cut-off value to distinguish NPC patients from healthy people. As shown in Figure 3B, MMP3 concentration can differentiate NPC patients from healthy controls with an AUC of 0.780 (95% CI: 0.663-0.846). Table 2 demonstrates that the sensitivity of MMP3 concentration was 74.8% and the specificity was 64.0% based on the optimal cut-off (29.84 ng/ml) according to the Youden Index. Moreover, the combination of MMP3 with the EA-IgA and VCA-IgA increased the value of AUC than the combination of EA-IgA and VCA-IgA alone (0.93* vs.* 0.89) (Figure 3C). In addition, the specificity of the combination of the three indicators was higher than the combination of the two EBV indicators (91.5% vs. 86.0%) without approximate sensitivity (Table 2). These results suggested that MMP3 protein level increases the specificity in NPC detection and offers additional diagnostic value in NPC.

### Diagnostic value of serum MMP3 activity in NPC

**Figure 4:**
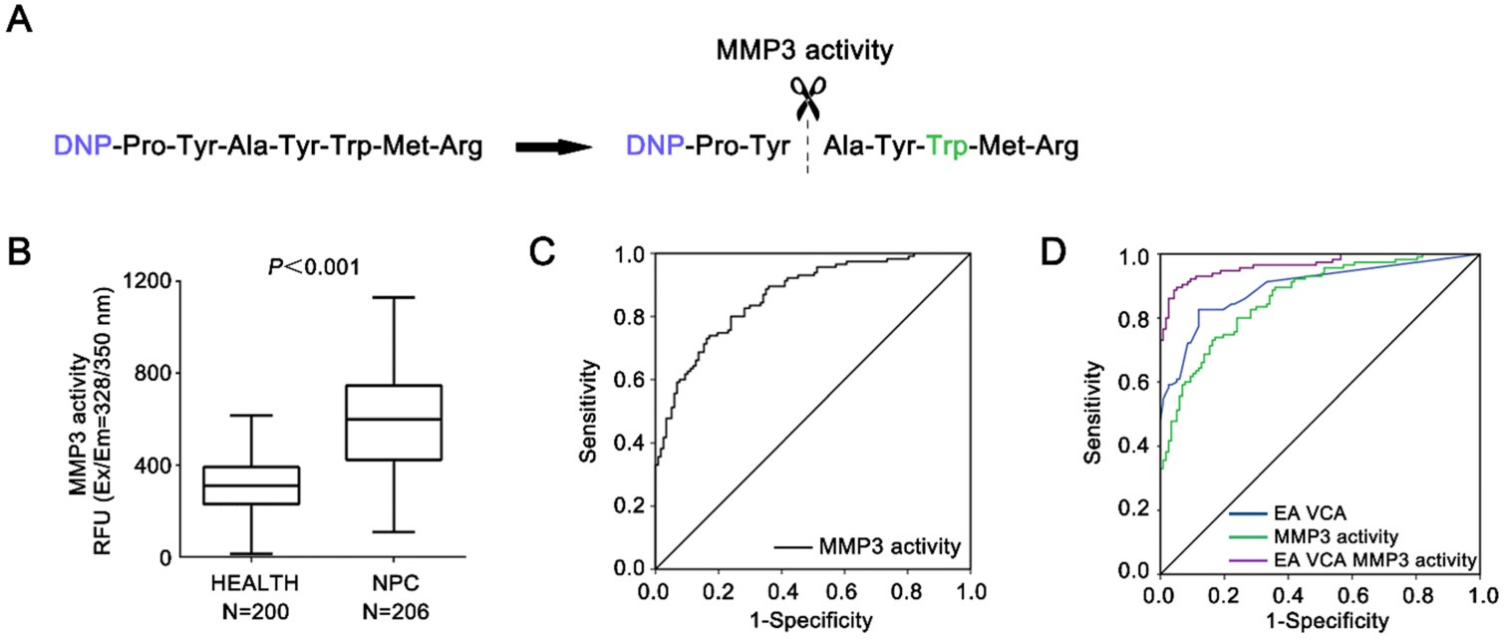
** Diagnostic value of serum MMP3 enzymatic activity.** (**A**) Schematic of detecting serum MMP3 activity with the FRET peptide of MMP3 substrate. (**B**) The serum MMP3 activity of NPC and control groups is plotted as a distribution. (**C**) ROC curves for the serum MMP3 activities. The estimated area under the ROC curve was observed as AUC = 0.86. (**D**) ROC curves for the diagnostic strength to identify NPC using MMP3 activity, EA-IgA and VCA-IgA (EA-IgA+VCA-IgA: AUC = 0.89; EA-IgA+VCA-IgA+MMP3 activity: AUC = 0.97).

The serum specific activities were also measured by MMP3 fluorescence-activated substrates in the same group of NPC patients and healthy controls, respectively (Figure 4A, B). The mean levels of serum MMP3 activity in NPC cases (601.4 ± 213.9 RFU) were significantly higher compared to control cases (308.5 ± 117.7 RFU, *P* < 0.001).

Furthermore, as shown in Figure 4C & D, the AUC for MMP3 activities was 0.86 (95% CI: 0.787-0.928) with an optimal cut-off value 389.2 RFU according to the Youden Index, whereas the AUCs for the combination of EA-IgA and VCA-IgA were 0.89 (95% CI: 0.81 8-0.953). Table 2 demonstrates the sensitivity and specificity of MMP3 activity were 80.0% and 76.1% based the cut-off value (389.2 RFU). Moreover, the sensitivity and specificity of MMP3 activities are both higher than that of MMP3 concentration (80.0% *vs.* 74.8%; 76.1% vs. 64.0%). By combining with the MMP3 activity, EA-IgA and VCA-IgA, the sensitivity and specificity reached 91.5% and 92.3% based on the optimal cut-off (AUC = 0.97). The sensitivity of the combination of EA-IgA and VCA-IgA with MMP3 activity was superior to that of the combination of MMP3 concentration (91.5% vs. 82.9%).

These findings validate the MMP3 activity as a serum marker to increase the diagnostic sensitivity for NPC, and indicate that the serum MMP3 activity and the EBV antibody combined have a better diagnosis values for screening NPC than traditional EBV antibody combined diagnosis.

### The association between serum MMP3 protein/activity levels and progression of NPC

The associations between the serum protein or activity levels of MMP3 and the clinicopathological parameters are presented in Table 3. The concentrations of MMP3 were not obviously correlated with any clinicopathological parameters including age, tumor size, T classification, lymph node metastasis, distant metastasis and clinical stage, but significant association was found with gender (*P* < 0.001). In addition, the MMP3 concentrations were not associated with the indicator of EBV infection, including levels of EA-IgA and ECV-IgA. In contrast, the serum MMP activity levels exhibited significant association with lymphoid nodal status (*P* = 0.043), clinical stage (*P* = 0.006) and distant metastasis (*P* = 0.024).

Those findings suggested that elevated levels of serum MMP activity, but not the concentrations were associated with NPC development and progression.

### The relationship between serum MMP3 protein/activity and EBV infection in NPC

**Figure 5:**
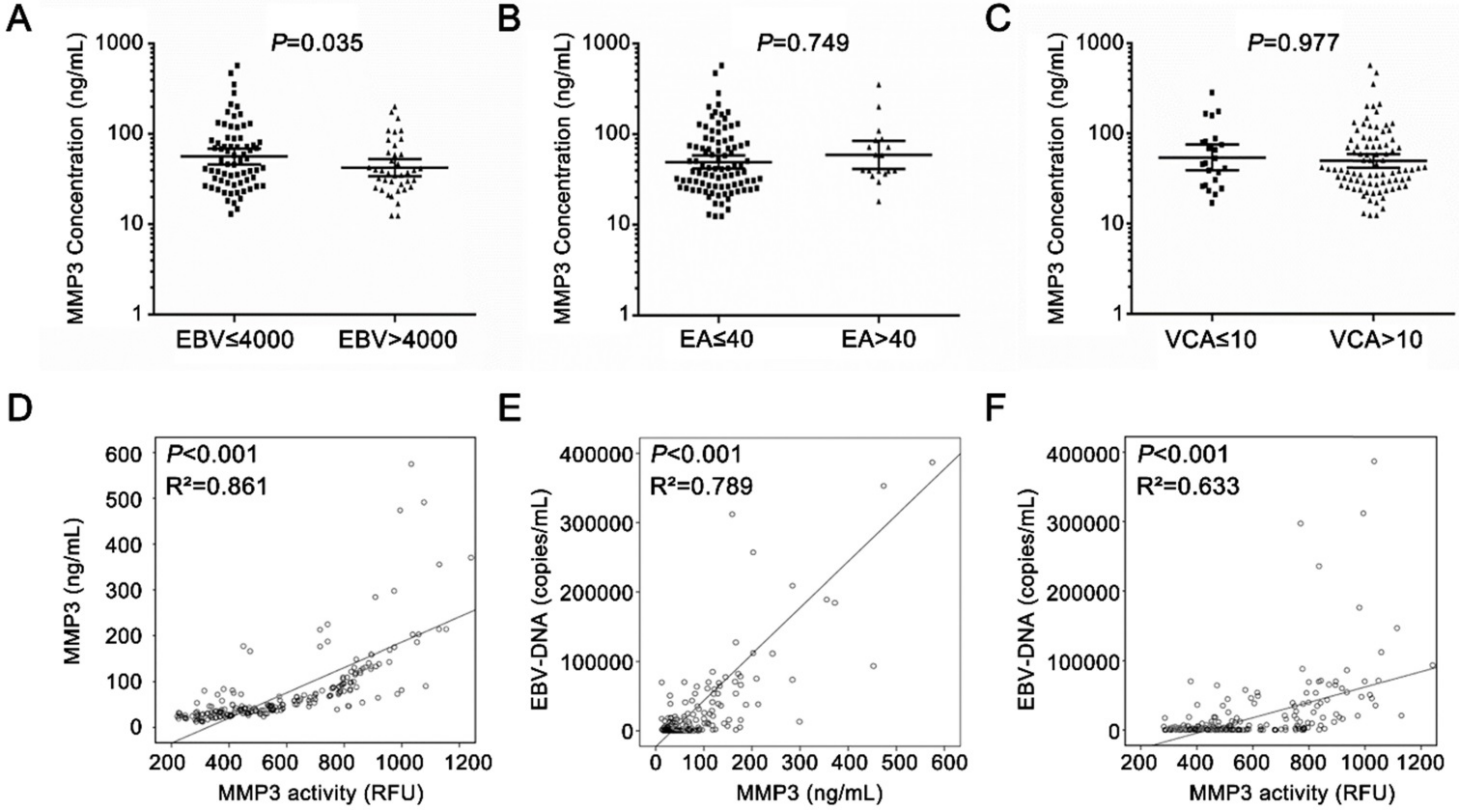
** The relationship between serum MMP3 protein/activity and EBV-DNA copies in NPC patients.** (**A-C**) The MMP3 concentration in EBV groups, EA-IgA groups and VCA-IgA groups. (**D**) Serum MMP3 enzymatic activities and concentrations were significantly positively correlated (*P* < 0.001, R^2^ = 0.861). (**E**) Serum MMP3 concentrations were significantly positively correlated with EBV-DNA copies in NPC patients (*P* < 0.001, R^2^ = 0.789). (**F**) Serum MMP3 enzymatic activity was significantly positively correlated with EBV-DNA copies in NPC patients (*P* < 0.001, R^2^ = 0.633).

The development of NPC is closely related to the infection of EBV 2. MMP3 level is associated with EBV infection. As shown in Figure 5A, serum MMP3 activities were significantly higher in the high EBV copies group (> 4,000) than the low copies group (< 4,000). However, there was no significant difference between in EA-IgA-positive group and EA-IgA-negative group (< 1:40 *vs* > 1:40, *P* = 0.794) in NPC patients (Figure 5B). Similar result was found in VCA-IgA-positive and VCA-IgA-negative group (< 1:10 vs > 1:10,* P* = 0.977) (Figure 5C).

Next, person correlation coefficient and a linear regression analysis were applied to analyze the correlation between MMP3 level and EBV infection in NPC patients. As shown in Figure 5D, the serum MMP3 concentrations were significantly correlated with the activity levels in NPC patients (*P* < 0.001, R^2^ = 0.861). As shown in Figure 5E & F, both of the serum MMP3 concentrations and activities were significantly positively correlated with the EBV copies (*P* < 0.001, R^2^ = 0.789; *P* < 0.001, R^2^ = 0.633).

Those findings suggested that EBV infection could be involved in the expression of MMP3 in NPC patients.

## Discussion

In this current study, we found serum MMP3 levels were significantly elevated in NPC patients compared to healthy control individuals. Moreover, we observed that MMP3 enzymatic activities were significantly associated with metastasis of tumor. Many of the members of MMPs family have been investigated as tumor markers, so we further explored the clinical significance of serum MMP3 in NPC.

Several studies have reported that the higher MMP3 expression in the stromal compartment compared to normal tissues 10,11,13. MMP3 is expressed primarily by stromal cells, including fibroblast cell and tumor-infiltrating macrophages. MMP3 has also been investigated to over-express in the tumor cell of tissues, including breast cancer, lung cancer, and pancreatic colorectal 14, and indicate poor survival in those tumor types. Consistent with those findings, our study revealed the strong expression of MMP3 in stromal and tumor compartments of NPC tissues. MMP3 protein was majorly located in cell membrane or distributed between the cytoplasm, but in the normal nonmalignant tissue or atypical hyperplasia adjacent to cancers, weak or no MMP3 staining was detected.

Furthermore, a panel of NPC and fibroblast cell lines was found high levels of MMP3 expression both in mRNA and protein. Meanwhile, higher levels of secreting MMP3 were also found in the cell supernatant of most NPC cell lines and fibroblast cell lines. Those studies gave a conclusion that both tumor and stromal of NPC tissues over-expressed MMP3, which contributed to the extremely high levels of circulating MMP3 in patients with NPC.

MMP3, as a secreted protein, its distinct serum levels made it a potential diagnostic marker of some diseases. Circulating protein levels of MMP3 have been reported to be increased in oral squamous cell carcinoma (OSCC), gastric cancer and prostate carcinoma 15. Moreover, serum MMP3 concentrations have been investigated to meet the criteria of a good diagnostic test for ovarian cancer 16. Recently, high levels of serum MMP3 concentrations were reported in NPC, which is consistent with our results 10. In our study, both of serum MMP3 concentration and activity exhibited the improved diagnostic ability for NPC detection. Besides, MMP3 activity showed both higher sensitivity and specificity than MMP3 concentration (80.0% *vs.* 74.8%; 76.1% *vs.* 64.0%). In addition, MMP3 activity, but not MMP3 concentration, was positively correlated with the progression of NPC. We found associations of levels of MMP3 activity with NPC patients' clinic stage and distant metastases. In line with our findings, a previous study showed that there is no correlation in serum MMP3 concentrations with clinicopathologic features such as tumor stage, tumor size, nodal status, and histological grade in OSCC 17. Those studies suggested that MMP3 enzymatic activity may be more valuable for diagnosis NPC than the protein concentration.

Currently, the measurement of enzyme activity in serum/plasma has been employed widely in clinical diagnosis 18-20. However, most of the research focus on the study of MMP3 concentration due to the convenient methods for the quantification of protein concentration by ELISA. The measurement of plasma/serum MMP3 activity had been explored limitedly for some diseases such as osteoarthritis 21 and Alzheimer disease 22. Previously, serum MMP3 activity has been monitored by MMP3 fluorescence-activated substrates which is highly specific for MMP3 and substantially not hydrolyzed by other MMPs 23. In our study, MMP3 concentrations only display moderate correlation with its enzymatic activities, due to the influence of the presence of inhibitors, cofactors, activators, unusual amounts of substrate or product in the serum 24, 25. Our results suggested that MMP3 concentration cannot completely reflect its enzymatic activity, and the survey of serum MMP3 enzymatic activity is worth exploring in those studies. Unfortunately, the diagnostic value of MMP3 for earlier patient appears insufficient (AUC <0.75), the more sensitive methods to detect MMP3 activity need to be improved in further study.

Tissue inhibitor of matrix metalloproteinase-1 (TIMP1), an endogenous inhibitor of MMP3, can inhibit the enzymatic activity of MMP3 potently. In addition, TIMP1 can promote cell growth and inhibit apoptosis by MMP-independent method 26. Numerous studies have shown that expression of TIMP1 is higher in either tumor tissue extracts or in blood from patients suffering from different tumor types 27-29. Interestingly, X-linked gene TIMP1 expression is from some, but not inactive X chromosome, suggesting that TIMP1 inactivation is polymorphic in human females 30. In our study, significantly higher levels of MMP3 concentration and enzymatic activity were observed from serum of female patients than male patients, indicating that lower levels of TIMP1 cannot effectively inhibit MMP3 enzymatic activities in female patients. Moreover, the mean levels of MMP3 protein in NPC patients increased about 6 times than healthy controls (185.97 *vs.* 33.95 µg/L), whereas MMP3 activities only increased about 2 times (601.4 *vs.* 308.5 RFU). Those results suggest that serum MMP3 protein plays its enzyme function by complex regulatory mechanisms.

Epstein-Barr virus (EBV) has been confirmed to be strongly related to the occurrence and progression of NPC. In our study, the EBV-positive C666 cell lines show higher expression and activity of MMP3 than the EBV-negative cell lines. Moreover, in MRC5 fibroblast cell line, we found that MMP3 expression level increased after coculturing with C666-CM for 72 h. These findings suggested that infection of EBV may increase the expression of MMP3 in cells. These results are consistent with a previous study showing that MMP3 was up-regulated by EBV Zta 31. In line with our study, other studies about virus infection and MMP3 expression display similar results. For example, HIV infection and HIV/HCV coinfection were identified to increase TIMP1 expression and suppress MMP3 expression in hepatoma and hepatic stellate cell lines 32. In addition, MMP3 has been shown to promote cellular antiviral response against Dengue virus infection 33. Those studies suggested the connection between circulating MMP3 levels and EBV infection may be more complicated than we already know.

Currently, EA-IgA and VCA-IgA are the two most commonly used diagnosis markers to monitor the progression of NPC 34-36. However, VCA-IgA had the highest sensitivity and limited specificity while EA-IgA had the best specificity and poor sensitivity 37, 38. It is expected that the multiple markers need to be combined in order to yield adequately accurate classification. In our study, MMP3 activity further combined with EA-IgA and VCA-IgA greatly improved the sensitivity and specificity (91.5% *vs.* 82.6%; 92.3% *vs.* 86%) for NPC detection. These results demonstrated serum MMP3 activity, better than MMP3 protein concentration, could be a potential biomarker for diagnosis of NPC, and suggested that the combination of MMP3 activity and EBV antibody exhibited potential value for screening and diagnosis NPC.

In summary, our study shows that the higher expression of MMP3 is observed in NPC tissues which may be the source of serum MMP3 in NPC patients. Moreover, we found out serum MMP3 level can screen and improve the diagnosis of NPC by combining with traditional EBV-infection biomarkers, which demonstrated that circulating MMP3 level, especially its enzymatic activity level, could be a useful biomarker to screen and diagnose of NPC. In addition, MMP3 is a member of extracellular proteases, which is also attractive drug targets, MMP3 inhibitor may be a potential therapeutic drug for patients with NPC. Our study also demonstrated that the MMP3-activated pro-drug or *in vivo* diagnostic probe, which could be degraded in peripheral blood, could be ineffective in the treatment and diagnosis for MMP3 over-expressed tumor types, such as NPC.

## Figures and Tables

**Table 1:** Clinical characteristic of 206 patients with NPC

Characteristic	No. (%)
**Sex**	
Male	153 (74)
Female	53 (26)
**Age**	
Mean	44.7
Range	16-75
<45	107 (52)
≥45	99 (48)
**Tumor Size**	
T1+T2	36 (17)
T3+T4	170 (83)
**Lymphoid nodal status**	
N0	17 (8)
N1-3	189 (92)
**Clinical Stage**	
1+2	31 (15)
3+4	175 (85)
**Metastasis**	
Yes	26 (13)
No	180 (87)

**Table 2:** The diagnostic value of EA, VCA, MMP3 and MMP3 activity (MMP3-A) in NPC

Combinations	Cut-off value	Sensitivity (%)	Specificity (%)
EA+VCA	0.48	82.6	86.0
MMP3	29.84	74.8	64.0
EA+VCA+MMP3	0.42	82.9	91.5
MMP3-A	544.40	80.0	76.1
EA+VCA+MMP3-A	0.38	91.5	92.3

**Table 3:** Clinicpathological associations of MMP3 expression levels and enzymatic activity levels

Variables	Cases (n)	MMP3 (ng/L) (Mean±SD)	Significance (P*)	MMP3 activity (CFU) (Mean±SD)	Significance (P*)
**Gender**					
Male	153	41.81±30.96	0.001**	488.9±174.0	< 0.001**
Female	53	84.10±74.29		625.8±216.5	
**Age (y)**					
<45	107	82.10±73.24	0.785	581.5±230.0	0.642
≥45	99	76.52±70.91		600.7±197.4	
**Tumor size**					
T1+T2	36	56.26±47.18	0.339	506.2±227.9	0.177
T3+T4	170	76.82±79.24		608.5±208.2	
**Lymphoid nodal Status**					
N0	17	65.75±48.28	0.884	515.1±228.3	0.043*
N1-3	189	85.89±73.52		595.1±213.8	
**Clinical Stage**					
1+2	31	49.59±38.85	0.285	396.7±69.3	0.006**
3+4	175	88.71±76.45		616.9±213.8	
**Metastasis**					
Yes	26	61.54±50.75	0.471	728.5±204.1	0.024*
No	180	74.55±87.75		575.1±210.8	
**EA**					
<1:10	38	78.32±67.69	0.768	623.2±230.0	0.454
≥1:10	168	72.09±54.33		583.3±211.2	
**VCA**					
<1:40	64	68.06±60.25	0.676	582.4±221.2	0.792
≥1:40	142	75.48±73.53		594.2±212.6	

## Abbreviations

NPC: nasopharyngeal carcinoma
MMP3: matrix metalloproteinase-3
VEGF: vascular endothelial growth factor
EBV: Epstein-Barr virus
EA: EBV early antigen
VCA: EBV viral capsid antigen
ROC curve: receiver operating characteristic
AUC: area under the curve
RFU: relative fluorescence units

## References

[1] Shin HR, Shin A, Woo H (2016). Prevention of infection-related cancers in the WHO Western Pacific Region. Jpn J Clin Oncol.

[2] Cao SM, Liu Z, Jia WH (2011). Fluctuations of epstein-barr virus serological antibodies and risk for nasopharyngeal carcinoma: a prospective screening study with a 20-year follow-up. PloS one.

[3] Ou Yang PY, Su Z, Tang J (2014). Diabetes, prediabetes and the survival of nasopharyngeal carcinoma: a study of 5,860 patients. PloS one.

[4] Kessenbrock K, Plaks V, Werb Z (2015). Matrix metalloproteinases: regulators of the tumor microenvironment. Cell.

[5] Gialeli C, Theocharis AD, Karamanos NK (2017). Roles of matrix metalloproteinases in cancer progression and their pharmacological targeting. Febs J.

[6] Bourboulia D, Stetler-Stevenson WG (2010). Matrix metalloproteinases (MMPs) and tissue inhibitors of metalloproteinases (TIMPs): Positive and negative regulators in tumor cell adhesion. Semin Cancer Biol.

[7] Roy R, Yang J, Moses MA (2009). Matrix metalloproteinases as novel biomarkers and potential therapeutic targets in human cancer. J Clin Oncol.

[8] Warnecke-Eberz U, Metzger R, Holscher AH (2017). Diagnostic marker signature for esophageal cancer from transcriptome analysis. Tumor Biol.

[9] Emily G, Kayla B, John W (2019). A pan-cancer perspective of matrix metalloproteases (MMP) gene expression profile and their diagnostic/prognostic potential. BMC Cancer.

[10] Gong DY, Li ZP, Ding R (2019). Extensive serum biomarker analysis in patients with nasopharyngeal carcinoma. Cytokine.

[11] Jin Y, Yang Y (2019). Identification and analysis of genes associated with head and neck squamous cell carcinoma by integrated bioinformatics methods. Mol Genet Genom Med.

[12] Finch-Arietta M, Johnson W, Lusch L (1993). Characterization of a tight-binding MMP-3 inhibitor using improved fluorescence spectroscopy techniques. Agents Actions.

[13] Ichinose J, Shinozaki-Ushiku A, Nagayama K (2016). Immunohistochemical pattern analysis of squamous cell carcinoma: Lung primary and metastatic tumors of head and neck. Lung Cancer.

[14] Mehner C, Miller E, Nassar A (2015). Tumor cell expression of MMP3 as a prognostic factor for poor survival in pancreatic, pulmonary, and mammary carcinoma. Genes & Cancer.

[15] Yeh YC, Sheu BS, Cheng HC (2010). Elevated serum matrix metalloproteinase-3 and -7 in H. pylori-related gastric cancer can be biomarkers correlating with a poor survival. Digest Dis Sci.

[16] Aneta CP, Anita CG, Ewa PS (2018). Suitability assessment of baseline concentration of MMP3, TIMP3, HE4 and CA125 in the serum of patients with ovarian cancer. J Ovarian Res.

[17] Andisheh-Tadbir A, Khademi B, Kamali F (2014). Upregulation of serum vascular endothelial growth factor and matrix metalloproteinase-3 in patients with oral squamous cell carcinoma. Tumor Biol.

[18] Alhusseiny AH, Al-Nimer MS, Al-Neamy SI (2015). Assessment of Serum Cystatin C Levels in Newly Diagnosed Acute Myocardial Infarction at the Onset and at the Time of Hospital Discharge. Cardiovasc Res.

[19] Scott DL, Wolfe F, Huizinga TW (2015). Rheumatoid arthritis. Lancet.

[20] Brennan ML, Penn MS, Van Lente F (2013). Prognostic value of myeloperoxidase in patients with chest pain. New Engl J Med.

[21] Kubota E, Imamura H, Kubota T (1997). Interleukin 1 beta and stromelysin (MMP3) activity of synovial fluid as possible markers of osteoarthritis in the temporomandibular joint. J Oral Maxil Surg.

[22] Peng M, Jia J, Qin W (2016). Plasma gelsolin and matrix metalloproteinase 3 as potential biomarkers for Alzheimer disease. Neurosci Lett.

[23] Finch-Arietta M, Johnson W, Lusch L (1993). Characterization of a tight-binding MMP3 inhibitor using improved fluorescence spectroscopy techniques. Agents Actions.

[24] Nagase H, Visse R, Murphy G (2016). Structure and function of matrix metalloproteinases and TIMPs. Cardiovase Res.

[25] Chakraborti S, Mandal M, Das S (2016). Regulation of matrix metalloproteinases: an overview. Mol Cell Biochem.

[26] Nagase H, Brew K (2013). Designing TIMP (tissue inhibitor of metalloproteinases) variants that are selective metalloproteinase inhibitors. Biochemical Society Symposium.

[27] Pesta M, Kulda V, Kucera R (2016). Prognostic significance of TIMP-1 in non-small cell lung cancer. Anticancer Res.

[28] Christensen IJ, Brunner N, Dowell B (2015). Plasma TIMP-1 and CEA as Markers for Detection of Primary Colorectal Cancer: A Prospective Validation Study Including Symptomatic and Non-symptomatic Individuals. Anticancer Res.

[29] Grunnet M, Mau-Sorensen M, Brunner N (2013). Tissue inhibitor of metalloproteinase 1 (TIMP-1) as a biomarker in gastric cancer: a review. Scand J Gastroentero.

[30] Anderson CL, Brown CJ (1999). Polymorphic X-chromosome inactivation of the human TIMP1 gene. Am J Hum Genet.

[31] Lan YY, Yeh TH, Lin WH (2013). Epstein-Barr virus Zta upregulates matrix metalloproteinases 3 and 9 that synergistically promote cell invasion *in vitro*. PloS one.

[32] Lin W, Wu G, Li S (2011). HIV and HCV cooperatively promote hepatic fibrogenesis via induction of reactive oxygen species and NFkappaB. J Biol Chem.

[33] Zuo X, Pan W, Feng T (2014). Matrix metalloproteinase 3 promotes cellular anti-dengue virus response via interaction with transcription factor NFkappaB in cell nucleus. PloS one.

[34] Zheng XH, Lu LX, Cui C (2016). Epstein-Barr virus mir-bart1-5p detection via nasopharyngeal brush sampling is effective for diagnosing nasopharyngeal carcinoma. Oncotarget.

[35] Tay JK, Chan SH, Lim CM (2016). The Role of Epstein-Barr Virus DNA Load and Serology as Screening Tools for Nasopharyngeal Carcinoma. Otolaryngol Head Neck Surg.

[36] Gu AD, Lu LX, Xie YB (2009). Clinical values of multiple Epstein-Barr virus (EBV) serological biomarkers detected by xMAP technology. J Transl Med.

[37] Sun P, Chen C, Chen XL (2014). Proposal of a clinical typing system and generation of a prognostic model in patients with nasopharyngeal carcinoma from Southern China. J Buon.

[38] Chen H, Chi PD, Wang WD (2014). Evaluation of a semi-quantitative ELISA for IgA antibody against Epstein-Barr virus capsid antigen in the serological diagnosis of nasopharyngeal carcinoma. Int J Infect Dis.

